# Pre-hospital intra-osseous freeze dried plasma transfusion: a case report

**DOI:** 10.1186/2054-314X-1-8

**Published:** 2015-03-25

**Authors:** Misgav Rottenstreich, Itzik Malka, Elon Glassberg, Oren Schwartz, Bader Tarif

**Affiliations:** 13 Trauma branch, Medical corps IDF, Tel Aviv, Israel; 14 Medical corps IDF, Tel Aviv, Israel; 15 The Institute of Research of Military Medicine, Medical corps IDF, Tel Aviv, Israel

## Abstract

**Background:**

Hemorrhage and coagulopathy are among the leading causes of death in combat and are considered the leading causes of preventable deaths. Plasma, in the form of Fresh Frozen Plasma (FFP) is considered a key component in the Damage Control Resuscitation performed within hospitals. Freeze-Dried Plasma (FDP) can be stored at room temperature and therefore is potentially useful in pre-hospital conditions. Our case report join to few cases where FDP was administered at the point of injury. It is also unique as it describes an intra- osseous administration given to pediatric patient.

**Case report:**

M.S. otherwise healthy 13 year old girl was injured due to gunshots and grenade blast. On the first triage by the IDF medical teams she suffered from: Severe hemorrhagic shock, (Blood pressure could not be measured, Heart rate 163), superficial wounds to her face, (forehead and Rt. Eye), gunshot wounds with active bleeding from her Lt. Arm and her RT. Knee (Mangled Extremity Severity Score (MESS) 8) and open fractures of left elbow and right thigh. A peripheral intravenous catheter was established and 1 g tranexamic acid in 500 ml of Hartman fluid were administered. Due to difficulties in establishing a functioning intra-venous line, an intra-osseous catheter was established and one unit of FDP (250 ml) was given in the field. She was transferred by a military medical team to a regional civilian hospital for further treatment. Upon arrival to the hospital her blood pressure and heart rate were significantly improved. After three weeks of hospitalization M.S. was discharged and she was returned to her homeland.

**Conclusion:**

We have described the successful use of FDP for pre hospital resuscitation of a 13 year old girl suffering from severe hemorrhagic shock as a result of gunshots and grenade blast. This case report demonstrates that intra-osseous FDP administration for as part pre hospital resuscitation of children has a favorable outcome.

## Background

Massive hemorrhage with coagulopathy is the leading cause of death in combat [1, 2]. Human plasma is considered the treatment of choice for severe coagulopathy [3–5].

Fresh Frozen Plasma (FFP) is widely used in the hospital settings for indications including severe coagulopathy and blood loss due to trauma. The advantages of the usage of plasma are: 1. Volume replacement 2. Preservation of intra vascular volume due to plasma proteins 3. PH balanced fluid 4. Replacing and assisting hemostasis by adding coagulation factors. However, the need for special conditions to store FFP makes it practically impossible to be used in pre-hospital conditions, especially in combat settings. Thus, crystalloids or colloids currently serve as the common first line for volume resuscitation in the pre-hospital arena [ref TCCC, ACLS [6]]. Freeze-Dried Plasma (FDP) can be stored in room temperature, making it suitable for use in the pre-hospital conditions. FDP was used during World War II by the American army and others6. However, due to the risk of infection transmission, especially hepatitis, its use was stopped at 1968. Single donor FDP was introduced in 2007 [7]. This product significantly reduced the risk of transmitted infection, by that, transforming FDP into a safer product [3]. Since, more than 230,000 units of FDP were administered in the hospital setting, mainly in Germany and France [3]. In vitro analysis of FDP has demonstrated a small decrease in factors V and VIII (ranging from a 25% decrease to no decrease) when compared with fresh plasma. The clinical effectiveness and adverse reactions of FDP were found to be very similar to FFP making it an appropriate alternative [3, 8, 9]. However, the use of plasma in pre-hospital settings is very restricted with a very small number of reports found in Pubmed search. These reports mainly describe the use of plasma in role 3 combat-hospital-facility or during helicopter transport of wounded to the hospital [1, 10]. The only previously reported use of FDP at the point of injury was published by the Israeli Defense Forces [11–13]. We describe administration of FDP to a 13 years old girl through intra-osseous catheter.

## Case report

M.S., a previously healthy 13 years old girl injured as a result of gunshots and grenade blast. She sustained injuries to her face and extremities. She was initially treated by a primary care physician of the IDF. The nearest hospital to the place of admission was a civilian hospital located about one hour drive away. While waiting for the ambulance for evacuation, resuscitation efforts were started. GCS was 15; she was restless due to severe pain. Systolic blood pressure could not be measured and the heart rate was 163 beats per minute. Her injuries included: 1. Active hemorrhage from open large wounds on the right knee (Mangled extremity severity score (MESS) 8). 2. Open fractures of left elbow and right thigh 3. Active hemorrhage from the left forearm and RT. Knee due to gunshot wounds. 4. Open superficial wounds on the forehead, right eye and face. Interventions included: Arterial tourniquet to the left forearm that was converted to hemostatic dressing during the secondary survey and a C-A-T tourniquet was applied to the right thigh. Dressing with antibiotic ointment to the wounds on her face was also applied. A peripheral intravenous catheter was established and adult dose of tranexamic acid (1 g) was administered in 500 ml of Hartmann solution. The IV line was pulled out by the restless patient and in order to assure a secure and rapid line for resuscitation a tibial intra-osseous catheter (B.I.G produced by PerSys Medical) was established. Due to severe hemorrhagic shock one unit of FDP (distributed as a powder, produced by LyoPlas in Germany) (250 ml about 5.5. ml/kg) was reconstituted and then administered. Intra-osseous Morphine was also administered for pain control. No colloids, or other blood products were administered until hospital arrival. No laboratory workup was performed until after arrival to hospital. Upon arrival to the hospital, oxygen saturation was 98%, the blood pressure was 106/80 and the heart rate was 127 beats per minutes. During her 3 weeks hospitalization surgical repairs of her open fractures were performed. Since her return to her homeland M.S. was lost to follow up.

## Discussion

Plasma is considered the best available resuscitation fluid in cases of severe hemorrhage in the hospital settings. FDP can be stored in room temperature making it feasible for use in the pre-hospital settings, as has been recently reported. Our case report describes FDP administration through intra-osseous catheter. To our knowledge, is the first case report of this practice. In previously reported cases of field use of FDP by the IDF, all cases presented with severe hemorrhage with deep hemorrhagic shock, and most of the cases ended successfully with life salvage with no adverse effects [13]. Based on the previously published data and our experience, usage of FDP in pre-hospital and at the point of injury may be encouraged. Intra osseous (IO) access represents a reliable alternative to intra venous access and is increasingly being used in the pre-hospital setting [14–18] and in the emergency department [19, 20]. Furthermore, the usage of intra osseous access is recommended by the European Resuscitation Council when intravenous access cannot be established within the first 2 min of resuscitation or is otherwise difficult or impossible [21], and by the IDF’s clinical practice guidelines following to failed I.V. placement attempts. Moreover, despite the theoretically risk of clots formation, by administration of low flow rates fluids, blood products can be safely administered through the intraosseous line both in adults and in children [22]. However, administration of FDP by intra-osseous catheter has not been previously reported [13]. Nowadays plasma is considered to be the best available resuscitation fluid, and mortality has been shown to be reduced as earlier as plasma is administered. The favorable result of our case report supports administration of FDP through intra osseous catheter and in pre hospital setting according to the right indications.

## Conclusion

We have described the successful use of FDP for pre hospital resuscitation of a 13 years old girl suffering from severe hemorrhagic shock as a result of grenade blast. This case report demonstrates that intra-osseous FDP administration for pre hospital resuscitation of children has a favorable outcome. Based on our experience with FDP, we recommend to continue using FDP as a resuscitation fluid in cases of severe hemorrhagic shock parallel with animal and human studies comparing the usage of FDP to other resuscitation fluids.

## Consent

Since the patient returned to her home at Syria and was lost to follow up due to the war state in Syria practically there is no way to contact her and more than that we have no way to identify her address. Therefore, we tried to avoid any detail that may personally identify the patient. Unfortunately this is the best we could do since informed consent in this case is practically impossible to obtain.
